# Understanding knee osteoarthritis from the patients’ perspective: a qualitative study

**DOI:** 10.1186/s12891-017-1584-3

**Published:** 2017-05-30

**Authors:** Victoria Carmona-Terés, Jenny Moix-Queraltó, Enriqueta Pujol-Ribera, Iris Lumillo-Gutiérrez, Xavier Mas, Enrique Batlle-Gualda, Milena Gobbo-Montoya, Lina Jodar-Fernández, Anna Berenguera

**Affiliations:** 1 Department of Basic, Evolutionary and Educational Psychology, Universitat Autònoma de Barcelona; Psychology Faculty, Building B. UAB Campus, Bellaterra, Barcelona, 08193 Spain; 2 Primary Care University Research Institute (IDIAP Jordi Gol), Gran Via Corts Catalanes, 587, àtic, Barcelona, 08007 Spain; 3 Universitat Autònoma de Barcelona, Bellaterra, Cerdanyola del Vallès, Spain; 4 Primary Care Centre Can Bou, Ciutat de Màlaga, 18-20, Castelldefels, Barcelona, 08860 Spain; 5 Primary Care Centre Amadeu Torner, Amadeu Torner, 63, l’Hospitalet de Llobregat, Barcelona, 08902 Spain; 6 San Juan de Alicante University Hospital; Rheumatology Unit, Ctra N-332, Sant Joan d’Alacant, Alicante-Valencia, 03550 Spain; 7 Psychology of pain and rheumatological diseases, Av. Presidente Carmona, 10 bis 1°A, Madrid, 28020 Spain; 8 Primary Care Centre Sant Ildefons, Avda República Argentiana s/n, Cornellà de Llobregat, Barcelona, 08940 Spain

**Keywords:** Lifestyle, Knee osteoarthritis, Qualitative research, Health coaching, Primary health care, Interviews

## Abstract

**Background:**

No studies of Health Coach Interventions for knee OA sufferers that include patients’ perspectives have been published. The study assesses current clinical practice and primary care professionals’ advice from the patients’ perspective, in order to obtain a participative design for a complex intervention based on coaching psychology. Moreover, wants to analyse the experiences, perceptions, cognitive evaluation, values, emotions, beliefs and coping strategies of patients with knee osteoarthritis, and secondly the impact of these factors in the Self-management of this condition.

**Methods:**

It is an interpretative qualitative study. The study included patients with diagnosis of knee osteoarthritis (OA) from 4 primary health care centres in Barcelona. A theoretical sampling based on a prior definition of participants’ characteristics was carried out. Ten semi-structured interviews with knee OA patients were carried out. A content thematic analysis was performed following a mixed-strategy text codification in Lazarus framework and in emerging codes from the data.

**Results:**

The results are structured in two blocks: Experiences and perceptions of informants and Experiences of knee osteoarthritis according to the Lazarus model. Regarding experiences and perceptions of informants: Some participants reported that the information was mostly provided by health professionals. Informants know which food they should eat to lose weight and the benefits of weight loss. Moreover, participants explained that they like walking but that sometimes it is difficult to put into practice. Regarding experiences of knee osteoarthritis according Lazarus model: Cognitive evaluation is influenced by cognitive distortions such as obligation, guilt, dramatization and catastrophism. Values: Family is the value most associated with wellbeing. Helping others is another recurring value. Emotions: Most participants explain that they feel anxiety, irritability or sadness. Beliefs: To some, physiotherapy helps them feel less pain. However, others explain that it is of no use to them. Participants are aware of the association overweight– pain. Coping strategies: The strategies for coping with emotions aim to reduce psychological distress (anxiety, sadness, anger) and some are more active than others.

**Conclusions:**

The study highlights that patients with knee osteoarthritis require a person-centered approach that provides them with strategies to overcome the psychological distress caused by this condition.

**Electronic supplementary material:**

The online version of this article (doi:10.1186/s12891-017-1584-3) contains supplementary material, which is available to authorized users.

## Background

Clinical guidelines for knee osteoarthritis (OA) recommend non-pharmacological first-line management which should include weight loss, healthy eating habits, physical activity (PA), Self-management of pain, information-education and orthoses [1]. In particular, they recommend the education of patients even if current evidence remains inconclusive [2–4]. Indeed, non-pharmacological recommendations frequently lack precision regarding contents, duration, intensity and frequency and might result in the suboptimal care of OA patients observed in several studies [4].

Based on personal living experiences with the disease, the interactions with health professionals and the treatments received, the informants have identified various elements to take into account in the design, contents and format of a new intervention for knee OA implemented in Primary Health Care [5]. The impact of knee OA can be profound on self-esteem [6], it limits daily activity, causes a feeling of loss [7] and decreases work productivity. These effects could be mitigated if the health services provided more information on the disease and on how to manage it self-efficiently [8]. Moreover, taking into consideration the beliefs and expectations of patients can contribute to improve the interventions [9].

The theoretical foundation of Health Coaching (HC) is linked to the theoretical-conceptual framework of behavioural change [10] and has been defined as a behavioural intervention to facilitate the establishment and achievement of health promotion objectives, modify behaviours, reduce harmful habits, improve self-management of chronic conditions and increase health related quality of life [11].

Health Coaching (HC) can improve quality of life, change attitudes, decrease unhealthy habits, treatment adherence, self-management and pain in chronic patients [12–19] and also promote healthy eating and PA [20]. In order to live with knee OA, patients use coping strategies that are determined by their values, emotions and beliefs. These categories define the key components that knee OA sufferers perceive as important for the design of a coaching intervention [5].

No studies of HC interventions for knee OA sufferers that include patients’ perspectives have been published. The Medical Research Council has established a methodology for the development of complex interventions that consists of several phases and uses qualitative and quantitative methods [21, 22]. The first phase of this methodology aims at identifying the contextual factors that shape the theories of how the intervention works and might affect implementation, the modelling process and outcomes.

The objectives of this study were:

1. To identify current practice and advice of primary care professionals from the patients’ perspective in order to achieve a participative design of a complex intervention based on coaching psychology
2. To further understand the experiences, perceptions, cognitive evaluation, values, emotions, beliefs and coping strategies of patients with knee OA and the influence of all these factors in the Self-management of this condition.

## Methods

### Design

We conducted an interpretative qualitative study [23] to further our understanding of the phenomenon as experienced by the individual living with knee OA. The study also looked for patient related factors that could facilitate or restrict the implementation of the intervention (acceptability, adequacy, feasibility, integration within other programs, location, schedule and duration).

### Conceptual framework of the study: Lazarus stress model

The Lazarus stress coping model [24, 25] has been used to understand stressful life events. This framework is adequate for our research when we consider knee OA as a potential stressful factor. The level of distress and wellbeing of people are determined by their coping strategies.

A growing body of studies stresses the importance of psychological factors in the pain process. The Lazarus stress model has been transferred to the chronic pain field to study these factors. This model suggests that when there is a potentially stressful event, anxiety levels depend on the cognitive evaluation of pain stimulus and the strategies used to cope with it. Psychological pain factors which have recently appeared in the literature are analysed from the perspective of the Lazarus model [26].

The Lazarus model includes the following variables:

- **Cognitive Evaluation:** process that determines the consequences that a particular event will generate in an individual.
- **Values:** Values convey what is important for the individual and determine what is at stake in a particular stressing situation.
- **Emotions:** Lazarus enumerates the following emotions: anger, envy, jealousy, anxiety-horror, guilt, shame, relief, hope, sadness-depression, gratefulness, compassion, happiness-joy, pride and love [27].
- **Beliefs:** cognitive configurations created individually or culturally shared. They are pre-existing notions of reality that can be used as a perceptual lens.
- **Coping Strategies:** constantly changing cognitive and behavioural efforts developed to manage specific external and/or internal demands that are evaluated as excessive or overwhelming for the resources of the individual.
- **Social Support:** coping resource whereby we have somebody that provides emotional, informative and/or tangible support.
- **Participants:** The study included symptomatic knee OA patients, with clinical and radiographic OA grades 1–3 in the Kellgren-Lawrence classification, selected from four primary health care centres (PHCC) in Barcelona. Participants were recruited by the general practitioners in each PHCC between February and April 2015.

### Sampling and participant selection strategy

A theoretical sampling based on a prior definition of participants’ characteristics was carried out to obtain optimal variety and discursive wealth [28]. Patients with knee OA living in the metropolitan area of Barcelona were selected in accordance to *a priori* defined profiles. We took the following variables into account: gender, age, number of years with knee OA, employment status, household and relevant health problems.

Patients with knee OA were recruited by their general practitioner or nurse, who explained the study and suggested the participation.

### Techniques to generate information

Face to face individual Interviews were conducted by the first author, a specialist in this technique, in the PHCC of the participants, and lasted from 30 to 60 min. The interview guide followed five sequences (impact of knee OA; interaction with health professionals, physiotherapists and social workers; coping strategy; recommendations toward the design of the intervention; and use of information technology techniques - ICTs) (Additional file 1). The interviewer used open, non-directive formulation consistent with the participant’s language. Observational field notes that included contextual characteristics, atmosphere and relevant non-verbal expressions were produced. Ten patients with knee OA were invited to participate, all of them accepted and data saturation was reached (Table 1).

**Table 1:** Sociodemographic characteristics of participants with knee OA

Code	Gender	Age (years)	Years since onset knee osteoarthritis	Employment status	Household size	Relevant health problems
M1_CR	Female	60	16	Housewife	Partner	Haemophilia and Depression
H2_CR	Male	84	14	Retired (building)	Partner	Silicosis, vascular problem in one leg
H3_CR	Male	58	20	Disabled (graphic design)	Partner	Pain in lower limbs
H4_SA	Male	85	6	Retired (factory)	Alone (widower)	Diabetes, bladder cancer (operated)
M5_U	Female	66	16	Disabled (cleaning)	Alone (divorced)	Polyarthritis, anxiety
M6_U	Female	62	15	Housewife	Partner	Polyarthritis, anxiety symptoms, high blood pressure
M7_U	Female	67	5	Retired	Partner	Knee prosthesis, carpal tunnel
M8_VR	Female	75	3	Housewife	Grandson	Knee prosthesis and breast cancer
M9_VR	Female	83	<1	Housewife	Alone (widow)	High blood pressure
M10_VR	Female	67	17	Retired	Alone (single)	Diabetes and depression

### Data analysis

All interviews were recorded and transcribed literally and systematically by trained personnel. After successive readings of the transcriptions, researchers reached preanalytical intuitions. A content thematic analysis was performed [24, 25]. Next, the following analytical steps were carried out: a) identification of the relevant texts; b) fragmentation of the text in units of meaning; c) a mixed-strategy text codification in Lazarus categories and in emerging codes from the data; d) creation of categories grouping the codes by the criterion of analogy; e) analysis of each category; and f) elaboration of a new text with the results. These results were subsequently discussed with the whole research team and, after careful deliberation and exchanges of findings, consensus was reached.

The following procedures were performed to improve rigour [29]: triangulation of analysis by four researchers; and comparison of findings with the original data.

## Results

The results are structured in two blocks: Experiences and perceptions of informants and Experiences of knee osteoarthritis according to the Lazarus model.

## Experiences and perceptions of informants

**Fig. 1:**
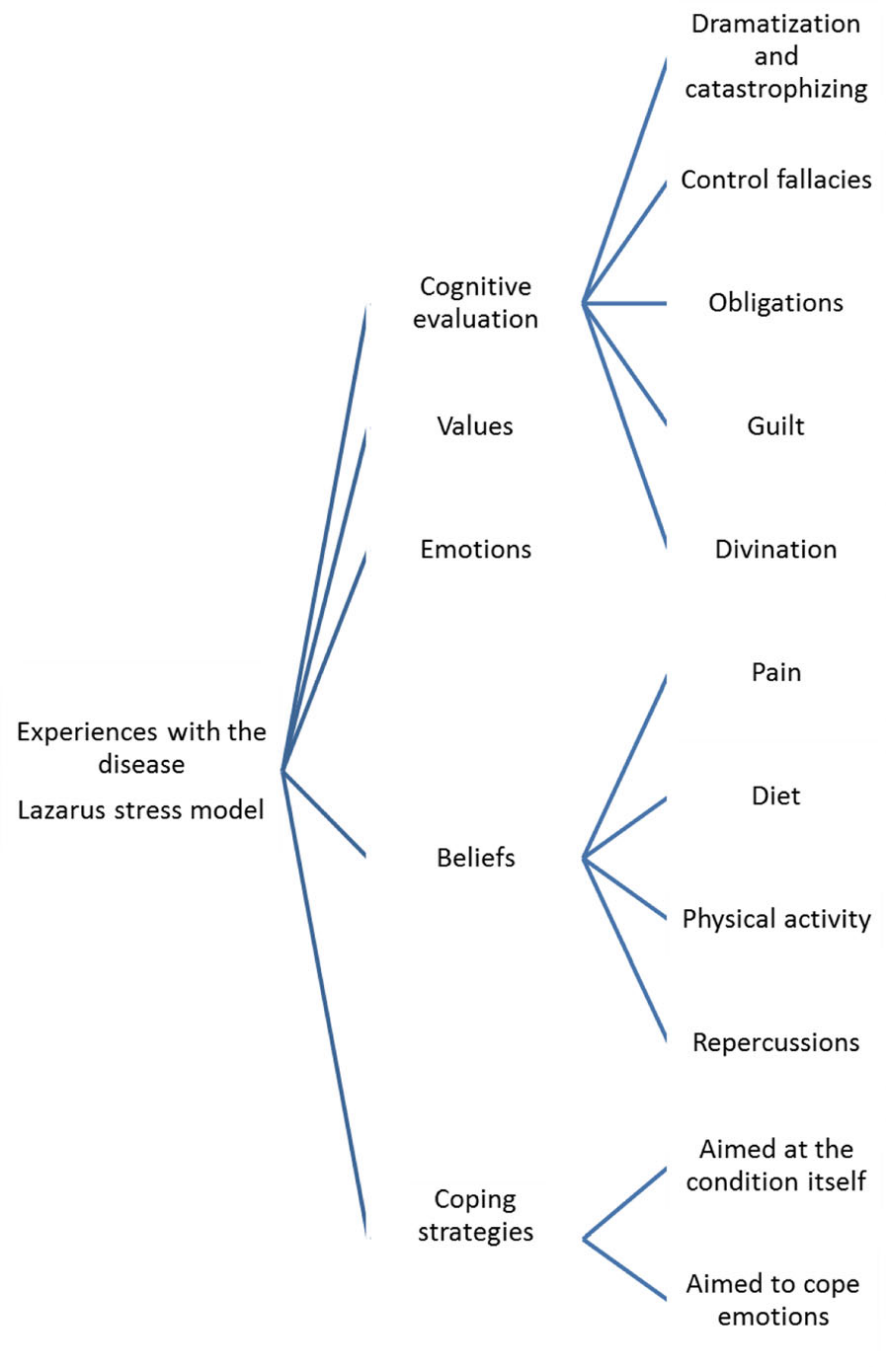
Lazarus theoretical model applied to knee osteoarthritis
  We have used the Lazarus Stress Model for a structured analysis of the patients’ experiences. This model explains that when faced with a potentially stressing event, people respond with different levels of stress and emotions. This response depends basically on two factors: cognitive evaluation (where according to Beck cognitive distortion takes place) and coping strategies. In summary, emotions depend on how the person evaluates the situation and which behaviours and thoughts does she use to manage this situation. However, this model also includes two additional cognitive variables of a more static nature that determine the other: beliefs and values. Consequently, based on their beliefs and personal values, individuals will evaluate and cope with a particular situation with different degrees of stress. An outline of the model can be found in Fig. 1.
  This model can be applied in this study, since knee osteoarthritis is a potentially stressing condition.
  A. ***Cognitive evaluation***
    Different cognitive distortions bias the cognitive evaluation of participants. For instance:
    Within the distortion “*obligations”,* the concept of helping those around them is the most used.
*“I have to do the shopping, I have to do the housework to my family” M7_U*
    In relation to *guilt*, they feel guilty about not following the advice on diet and PA and for not meeting the expectations of the people closest to them.
*“I feel bad when I eat something forbidden”. M6_U*
*Dramatization and catastrophizing* are observed when participants refer to pain and they label it as terrible, as greatly affecting them, etc.
*“… Terrible, terrible. But in 1998 it was less painful, I think that because I still had my house and was busy with my children, but since I came here, in 2001, uyy, this has been terrible” M10_VR*
    The *control fallacies* are conveyed in verbalisations such as lack of time or putting on armour to protect themselves from being hurt.
*“ until now I went swimming, but lately I had to quit due to family problems, I don’t have time ” “I know many thin women that have bad arthritis” M6_U*
    The *divination of thought* usually focuses on the feelings of the loved ones (they think they know what their family members think about them).
*“… For me it’s not a trauma or anything, people should see that … people are very nosey, they like to ask; there she is with the bad knee.. you have to explain, otherwise …” M1_CR*
  B. ***Values***
    Values are compromises that express what is important for the person. Family is the main answer to the question of what makes them happy and the value most associated with wellbeing. *Helping others* is another recurring value.
    Some of them prioritize the needs of others over their own needs, which they associate with the importance of the family.
*“And most of all not being in much pain, because I have things to do, … help my daughter, … help my mother…”. M1_CR*
    They lose sight of self-care. Another important value is *filling time*.
*“Being busy the whole day!! I spend cleaning … cleaning here and there… It does not even hurt.. M10_VR*
    The absence of “occupation” takes them back to dejection and sadness thoughts and emotions and feelings of uselessness.
    The value of *autonomy*, being physically independent and not needing canes and crutches; it is essential to help the family, others and to be busy.
*“It makes me sad because it limits me, it limits me a lot, I cannot do what I would do”. M8_VR*
    All participants mention self-demand (overcoming personal frontiers) and discipline by action (following medical recommendations) or by omission (neglecting to follow these recommendations). They consider themselves demanding in relation to their values and to the important aspects of their life. Prevention of knee OA was not considered a priority.
  C. ***Emotions***
    Most participants explain that they feel *anxiety, irritability* or *sadness*. Anxiety affects them differently: in some the diet is affected, in others it increases ruminative thoughts. In most cases, and even if not directly articulated, the low mood status is enhanced by the lack of acceptance of the aging process. They feel nostalgia for the activities they could do “before the arthrosis”, which in fact were activities they carried out in their youth. Indeed, knee osteoarthritis represents a “mourning process” for the participants.
*Fear* takes place when someone in their immediate environment had been seriously disabled as a consequence of osteoarthritis. In these cases, we observed a keener interest for prevention.
*“I had my mother sitting in a chair for many years as a result of arthrosis. That’s what I fear most” M8_VR*
    Participants find *happiness* in small things related to their values: housework, since it signifies *autonomy*; helping others; and spending time with the family.
  D. ***Beliefs***
    Beliefs are cognitive configurations individually created or culturally shared, pre-existing concepts of reality that act as a perceptual lens.
***Beliefs on pain***
      To some, physiotherapy helps them feel less pain. However, others explain that it is of no use to them.
*“No… Because when I’m doing physiotherapy it hurts even more, and when physiotherapy finishes it goes on hurting. It doesn’t work for me”. M1_CR*
      Patients believe that physiotherapy is useful when they are doing it and are aware that when they stop physiotherapy the pain comes back. Only one participant explained that he consistently practices the exercises daily because he believes that it is key to improve pain. For some, the pain of knee OA hinders the practice of physical activity because of the belief that it can increase the pain.
      Another shared belief on pain is “that it must be borne”, possibly related to some Judeo-Christian values of sacrifice, be it as acceptance (coping strategy) or some resignation (negative emotion).
      To cope with pain, patients choose as a first option looking for distractions, secondly they prefer to bear it and lastly they take medication.
***Beliefs on diet***
      Participants are aware of the association overweight– pain. The impossibility of losing weight is one of the most common beliefs amongst women with knee OA. They attribute this impossibility to menopause, age and other factors unrelated to the quality and amount of food intake. Anxiety surrounding food is another factor related to the difficulty in losing weight. Most participants believe that the dieting recommendations are not effective for their particular case. They pointed out that the leaflets with standard advice are of no use to them.
*“He gave me some paper, but I don’t follow it because it’s bad for me. However, him and another one have insisted that I should not put on weight ” M5_U*
***Beliefs on PA***
      Participants believe that PA is positive. When they are more active they feel better and their mood improves. The most common PA are walking and Aqua gym.
*“In water you can do any exercise” “outside water no”. M1_CR*
      The main limitations for being physically active were knee pain and in some cases negative emotions.
***Beliefs on OA***
      Participants explain that OA is a chronic disease and that professionals have told them that it cannot be cured*.* Beliefs on the possibilities of a cure for knee OA emerge in the interviews, whereas at a conscious level participants are aware that no cure exists yet. However, they mention hope and the need to find a curative treatment.
*“I think that everything but death has a cure”. M10_VR*
      Patients need to translate the medical indications into something practical in their daily lives. Resignation when faced with knee OA has a strong emotional impact and generates sadness, anger and anxiety. Some patients talked about acceptance with a resigned face and non-verbal signs of negative emotions.
  E. ***Coping strategies***
    The coping strategies aim either at emotions or at the condition itself.
    The strategies for *coping with emotions* aim to reduce psychological distress (anxiety, sadness, anger) and some are more active than others. Amongst the most active we highlight looking for distractions, for social support (more emotional than practical). Other patients use strategies to avoid thinking about their problem and being surrounded by family to feel better (one of the most commonly used strategies) such as praying or counting to 10 to calm down.
*“I spoil myself with a latte”. M8_VR*
*“I wake up around 10 a.m. First thing I drink lukewarm water with lemon, eh, I have breakfast after half an hour, and I make my beds, clean the floor, sweep the apartment, I do, I prepare lunch.. and in the afternoon I get out. ” H3_CR*
*“Mm.. little things, then you understand, how can I explain it.. look, right now I’m enjoying the tablet and my husband with the ball and me with the tablet is no problem at all ” M6_U*.
*“I get it out of my mind” (referring to the disease of the daughter). M8_VR*
    The most commonly reported strategy is acceptance or resignation.
*“If you accept it your attitude changes” H3_CR*
    Whereas for some acceptance is a negative concept, others assert that when you accept your condition you change your attitude for the better.
    The strategies for the condition (chronic and without a lasting solution) aim at reducing the deterioration and the pain (taking medication, walking, cold, rubs, rest and exercise.
*“When it’s very painful I take ibuprofen or naproxen”. M7_U*
*“Walking at my pace is very relaxing for me”. H3_CR*
*“And I rub it with wasps’ poison” M8_VR*
*“ if you join a gym, whether you believe it or not it’s super positive for the brain ” M10_VR*)
    They learn to pace activities so that the pain does not increase, and to “say no” to avoid being overloaded with obligations. Not all strategies are equally used. Some patients never learn to say “no” or to pace efforts. The strategies aimed at the condition can also be used for the emotions (the calming effect of exercise).

1. ***Experiences and perceptions in patients’ interactions with health professionals*** **(General Practitioner and nurse), physiotherapists and social workers** The following subcategories emerged from the informants’ discourses:
***Information-education on knee osteoarthritis***
    Some participants reported that the information was mostly provided by their family doctors, who explained the repercussions of knee OA, the progression of the disease and also provided some advice. However, a participant told that sometimes she did not understand the information provided. Others were not satisfied with the information received, in particular by specialists; they considered that it amounted practically to nothing and that the interaction was limited to prescribing painkillers and to referring them for diagnostic tests.
*Do you feel that the professionals of the health centre have provided enough information on knee osteoarthritis?. . No, they just prescribe painkillers. And I cannot take strong painkillers because I have a large hernia and they prescribe paracetamol, which is useless. M9_VR*
    None of the participants received materials on these issues, and some said it would be useful for them to have this information *although they did not specify which type of materials they expected. The patients’ expectations aimed to obtain more information on their condition, prognosis and treatment”.*
***Professional advice on diet and weight***
    Advice on losing weight was mentioned by most participants. Informants are aware that they are overweight, they know which food they should eat to lose weight and the benefits of weight loss. Some explained that they take care of their weight, but that putting on weight comes very easy to them. They find it difficult to modify behaviours, mainly because of lack of discipline and because of the food they love. They emphasized the difficulty of sustaining a healthy weight, they had lost weight with the help of dieticians, endocrinologists and acupuncture and have again put it on.
*“…I’m very strict …You tell me “take this list: Monday this, Tuesday that,..” and I will do it… don’t give me anything ambiguous for me to plan between 5 possible first courses, 5 possible second courses.. no. H4_SA*
    Some had received leaflets about diets to help them achieve weight loss.
***Professional advice on physical activity***
    Participants explained that health professionals tell them to walk. They explained that they like it and they walk even if it is difficult, because if they move they feel better.
    Some receive positive feedback for exercising in water to reduce impact on the joints,
*“Aguagym? Yesyesyes, it’s wonderful. I go twice a week, and then I feel so relaxed, because I leave the swimming pool, I have a shower…and such, I then feel so relaxed …” M1_CR*
    and others use the stationary bicycle. They explained that they would feel more motivated if they could exercise in a group.
***Experiences with the different treatments provided***
    Informants mentioned painkillers and non-steroidal anti-inflammatory agents, usually prescribed by health professionals. They are anxious about the side effects of these medications.
*“I avoid taking pills…I have some, in case one day it hurts too much….” M5_U*
    Depending on the situation, they choose to take paracetamol, NSAIDs, sometimes with a “gastric protection agent”, or bear the pain without taking any tablets.
    Gels and creams are well accepted by participants, but they complained that the public health system does not cover this type of treatment; they also complain about (the) long waiting times for local injections.
    Some participants had also had physiotherapy with mostly positive results on the short and medium term. In general, they complained about the long waiting lists to access these services.
    One informant explained a positive experience with magnesium supplements. They did not comment on the opinions of professionals on alternative therapies.
    Two participants have a knee prosthesis and they evaluate them positively. Most consider that prosthesis is the best option in the long term although they leave it for when their condition is unsustainable. The youngest participants explained that in this case the health professionals recommend to wait and to bear the condition. Two participants expressed the need for research in this area.
*“in November 2011 they were about to give me a prosthesis, I had the day of admission, for the operation and everything, but in the preoperative stage, the last person you talked to is the anaesthetist, and he really scared me he told me you are very young and the average duration of the prosthesis is 10 years, and there is more rejection in the second…and I didn’t do it,” H3_CR*
*“Well, if some people need the operation, they don’t want to suffer more then, explain, tell him to not be afraid, that the operation no no… My experience of the operation is very positive…” M8_VR*
2. **Experiences of knee osteoarthritis according to the Lazarus model (Fig.** 1 **)**

## II-Recommendations for the design of the coaching psychology intervention (Table 2)

**Table 2:** Recommendations for the coaching intervention – barriers and facilitators

Barriers in the management of knee osteoarthritis	
Result	Contributions
Difficulty for translating theory into practice	Strategies and techniques to take action
Difficulties in sustaining a healthy diet	Indications and recommendations for a healthy diet
	To facilitate simple menus
	To facilitate healthy menus
	Techniques to control stress (anxiety)
	Motivation strategies to implement a healthy diet and make it sustainable over time
Pain associated with physical activity	Techniques to manage pain
	Indications and recommendations to carry out physical activity
	Motivation strategies to start and sustain physical activity
Fear	Strategies to overcome fears
Facilitators in the management of knee osteoarthritis	
Result	Contributions
Holistic Vision	Holistic vision focused on individual needs
Lack of individualisation	Taking into account the person within the group
Physiotherapy to reduce pain	Include physiotherapy exercises in the intervention
	Strategies to sustain the practice of these exercises
They would like to have more information	To provide information on the disease, its repercussions and advice on how to live with it
Learning to say “no”	Assertiveness strategies
Social support	To offer space for participants to talk about their particular situation
	To offer the possibility of continuity of contact between participants at the end of the intervention (WhatsApp, phone, etc.)
Self-care	To provide strategies for the patient to have time for him/herself
Learning to calm down	Strategies to control stress
	Mindfulness
	Breathing and relaxation techniques
Acceptance	Work with acceptance and differentiating it from resignation
Values	The patients’ most important values will be used to motivate and to generate change in the participants (family, autonomy, discipline)

### *Reference framework*

Participants pointed out that even within a group intervention there is a need for a holistic, person-centered approach that does not just focus on arthrosis. They underlined the community aspect and the need to be made aware of the resources of the neighbourhood.

“*To help us know what’s in there, in this neighbourhood there is nothing to do. Things like aqua gym and such” M7_U*

On the other hand, they highlighted the relevance of a participative design that allows sharing experiences and improvement proposals. In their opinion, sharing makes people feel more involved in the activities and it implies more consistency in relation to learning and implementing what they have learned. However, some participants think that they might feel uncomfortable within a group. They also mentioned the importance of working with cultural and socioeconomic sensitivity.

Participants accepted and were open to the proposal of having a health psychologist as coach because they believed it is a professional trained to listen to and solve needs related to their disease.

*“I’m happy, because it’s working well with the doctor, and now with you, and if there are things to do for no, to slow down a bit the arthritis, then fantastic…·”. M7_U*

None of the participants manifested that they wanted another type of professional to conduct the Health Coaching.

### Contents/components

Participants emphasized the need to translate theory into practice for behavioural change. In general, they proposed to encourage the strengths of individuals and to promote strategies to live with a more positive, problem-solving attitude.

“*We need to cheer up people that feels down, teach them that they have to move even if they are in pain. Because I also have pain, eh?” M8_VR*

Participants also expressed the need to learn to say “no” to be able to focus on self-care. In relation to diet, they explained that they need to learn how to prepare restrictive diets and how to combine foods with nutrients. In relation to PA, they wanted to know the activities most suited to their financial, practical and health needs. With regard to pain, they wanted to know how to channel thoughts toward more positive attitudes.

### Use of TICs

Most participants mentioned the regular use of smartphones, tablets and computers. They knew how to use messaging apps and were open to work using these technologies.

“*Whatsapp yes. My friend in Italy, I will show you, that I have a friend in Italy, that friend I mentioned from Italy. Whatsapp yes and gmail too… Let’s see, Nicoletta… Every day or every night, “good night”, “”, “bon giorno” “#italiano#” “#italiano#”. M5_U.*

### Location, schedule and duration and frequency

In relation to location, they consider that activities should take place in any community site that is close and accessible (libraries, schools, Primary Health Care Centre, community and sports centres, parks). Moreover, with the use of different locations more people gets to know the resources and participation is enhanced.

With regard to schedule, they point out that arrangements are generally more difficult for working people, more so since the financial crisis. They suggest schedules that include morning and afternoon. As regards duration and frequency, they propose weekly frequency during 5–6 weeks, duration around 2–2.5 h.

## Discussion

In relation to interaction with professionals, participants acknowledged that even though they received advice on health behaviours for the management of knee OA and other conditions, adherence to this advice is difficult. They expressed fear and ambivalence in relation to surgical and pharmacological treatments and suggested that professionals should focus on the needs of the patients, on alleviating pain and on delaying the progression of the disease. These results coincide with various studies that indicate that patients and health professionals felt that OA should receive more attention and better consistency of care, with more emphasis on self-management to help patients manage their condition more effectively and appropriately [8, 30]. Indeed, patient information and education are considered core treatments for OA in evidence-based clinical guidelines. Patients should then use this information to implement changes and for decision-taking regarding treatment [31–34].

Similarly to other qualitative studies, patients explained that knee OA affects the whole body and their lives on many levels [35]. The symptoms experienced illustrate a diversity of problems that represented a major concern for many participants in our study: knee pain, stiffness, difficulties in daily activities, reduction of work, instability, weakness, lack of mobility and psychological impact. Patients with knee osteoarthritis often suffer from obesity/overweight (90%), hypertension (40%), depression (30%) and diabetes (15%), resulting in a decreased quality of life [36]. However, most patients can differentiate when the pain is due to knee osteoarthritis or other conditions.

With regard to psychological impact, we should highlight that most questionnaires that evaluate knee OA and its treatment do not include this factor, probably because there was no involvement of knee OA patients in its design [34]. In this study, participants mentioned the need for research in this area. Indeed, research projects should plan their agendas working with those that should benefit from that research [33]. Since they are best placed to reflect on their experience of an illness, it is essential to collect their opinions to build evidence about their priorities [37] and to enhance the relevance of research. A strength of our study is that we take into account patients’ perspectives. The study protocol [5] explains that the integral management of the patient is paramount in the treatment of knee osteoarthritis. Accordingly, the design of the coaching intervention takes into account other factors such as associated diseases, environment and personal circumstances, all of which are considered during the sessions.

Patients explained that translating theory into practice and modifying behaviour is difficult. They request a holistic vision from professionals, based on their needs and not only on their health problems [38]. The person-centered approach model involves being an active agent for change and a non-directive role of the professional; it is based on unconditional acceptance, empathy and authenticity [39, 40]. Including the patient-centered context in complex interventions implies reflection on how to support optimal health and provision of care by means of reflecting on the patient’s history [41].

The Lazarus [24, 25, 27] model used in the analysis is useful to detect cognitive evaluations and stress coping strategies. Acceptance is one of the key facilitators for coping with different situations [42–44] such as knee osteoarthritis. Working toward acceptance through HC can help patients improve the self-management.

Our findings point at the various difficulties for participants to follow dietary advice: lack of individualisation, anxiety, and prioritizing other aspects of their lives, in particular the people closest to them [30]. Some studies have shown that HC helps people achieve dietary objectives [45–47]. Also in agreement with other studies and despite a positive attitude in relation to PA, we have found barriers to their implementation such as pain, PA habits prior their current condition, lack of social support and lack of acceptance to their current situation [48–50]. According to various authors, HC provides strategies to start and sustain PA levels appropriate to the conditions of individuals [19, 51–56]. Another main result of our study is the importance that participants attach to mood, a key element for setting in motion their internal resources, also observed in previous investigations [34, 57–59]. In summary, health coaching, which originates within the framework of behavioural change, can help people increase their quality of life, their self-efficacy (a determinant factor of behavioural change), their perception of self-control and to reduce stress [30, 60].

### Strengths and weaknesses

This research investigates the experiences of patients with mild to moderate knee OA. These experiences constitute an essential information toward the design and implementation of a complex intervention that will be acceptable, adequate, feasible, integrated within other programs, and appropriate in relation to location, schedule and duration. This article corresponds to Phase 1 or Modelling Phase of the MRC framework; the results have been used to design a HC intervention in knee OA intervention [61, 62].

Most participants in our research have suffered from knee OA for long time, are extremely familiar with this condition, understand available interventions and resources and have developed effective coping strategies. Participants spoke extensively about their problems, so that the data obtained were rich and diverse. The theoretical sample was successful in achieving diversity in age, gender, number of years since onset of disease, employment status, treatment modalities and multimorbidity. We should underscore that the mean age of participants in this study is older than the mean age found in the literature. This is a qualitative study that aims to achieve maximum plurality of discourse (minimum age 58 years, maximum age 85). Although the average age in experimental studies on knee osteoarthritis is 62 years of age, the electronic medical records of primary care centres in Barcelona reveal that the mean age of most patients that consult for this condition is closer to 70, the age group most represented in this study.

Nevertheless, the results of our study have been limited because no adults in the pre-diagnostic phase were included and the immigrant population, which might have different experiences, was not represented. The coaching group sessions with a participative strategy could be useful in case of proactive participants and volunteers but not for other profiles. This is a common characteristic of many intervention studies that require voluntary participation. This issue is worth exploring in depth during a qualitative study (phase 3). Further studies should include their discourses. Therefore, caution is needed before transferring these results to other settings and populations. However, the similarity with other studies with people from other origins and cultures suggests its applicability [7–9]. The rigour procedures used (in-depth-description of the context, theoretical sample design, audio-taping transcription, saturation, triangulation of analysis and reflexivity) have ensured the reliability of the findings in our setting.

## Conclusions

This study analyses the experiences of patients that suffer from knee OA. Their discourses have been extremely useful for the modelling of the second phase of this study, a clinical trial to compare current treatment with the HC intervention.

Amongst the findings we should highlight the need for a holistic, person-centered approach that takes into account the characteristics and needs of patients and that includes the physical, psychological, social and practical aspects of knee OA.

These patients need empathy in relation to their suffering and psychological distress caused by their condition. They also explain their concern and ambivalence in relation to the use of medicines to control pain and request more research to obtain pharmacological treatment that is more effective and produces less side effects.

Health coaching should emphasize acceptance as a coping strategy. Acceptance is a key factor toward the improvement of quality of life and to decrease the impact of knee OA.

## Additional file

Additional file 1: Interview guide. (DOCX 16 kb)
