# Supplementary material for: Understanding knee osteoarthritis from the patients’ perspective: a qualitative study
Source: BMC Musculoskelet Disord. 2017 May 30;18:225. doi: 10.1186/s12891-017-1584-3 (PMC5450398; doi:10.1186/s12891-017-1584-3)
Supplement: Additional file 1: — Interview guide. (DOCX 16 kb) [file 12891_2017_1584_MOESM1_ESM.docx]

**Additional file 1:**

**Interview guide**

| ***We would be grateful if you could tell us about your opinions and experiences on:*** | |
| --- | --- |
| **Impact of knee osteoarthritis** | *What is the impact of knee osteoarthritis in your everyday life?* |
| **Relationship with health professionals in the treatment for knee osteoarthritis** | *Do you feel well informed about knee osteoarthritis? Where have you obtained the information from?* |
|  | *How often do you attend your primary care centre due to knee osteoarthritis?* |
|  | *Could you describe which therapeutic recommendations does your GP provide?*  *In addition to medicines, does your GP recommend weight loss and physical exercise?* |
|  | *What are the main difficulties for you to follow the therapeutic recommendations given by primary care professionals?* |
|  | *What else do you think the health professional can do in relation to knee osteoarthritis?* |
|  | *Does the health professional provide you with support information with regard to management recommendations for knee osteoarthritis?* |
| **Patient’s actions to minimise the effect of knee osteoarthritis** | *Which activities or strategies help you in your everyday life to live with knee osteoarthritis?* |
|  | *Which health prevention activities (physical activity, diet, pharmacological treatment) do you find easier to follow up? Which do you find more challenging?* |
|  | *In case you have achieved some change related to physical activity or diet: Has somebody helped you? How have you achieved these changes?* |
|  | *Which beliefs/expectations do you have on knee osteoarthritis: Do you think it can be cured or improved?* |
|  | *What do you think you can do to improve your condition* |
| **Relevant aspects to consider in the intervention for knee osteoarthritis** | *In the design of an intervention to help people with your condition: which key aspects would you highlight for a successful intervention on knee osteoarthritis? (at an individual, family and community-social level)* |
|  | *Do you have somebody to talk about your problems? Do you find it difficult to socialise? Do you prioritise other people’s needs before yours? Can you say no?* |
|  | *What is your relationship with your thoughts? Are they too circular, piercing and you don´t know how to stop them? Have you been stuck on some issue for a while? Do you ask too much of yourself? Do you carry with you some resentment that you have never forgiven? Do you find it difficult to forgive?* |
|  | *What do you do to be happier? Do you have time for things that you are excited about? What do you do to relax? Do you self-indulge? What are the small things that make you feel good? Which of your traits and behaviours should you change to be happier? Can you plan your time?* |
|  | *How does osteoarthritis influence your mood?* |
|  | *How would you plan the intervention so that you could attend? Location, days, hours, duration, follow-up.* |
| **Population and use of ICT** | *Do you have a mobile phone, WhatsApp, internet or social networks?* |
|  | *Would you like to receive text messages to support healthy behaviours?* |
